# Supplementary material for: Active Particles in Tunable Compressible Environments
Source: Small Sci. 2026 May 7;6(5):e202500599. doi: 10.1002/smsc.202500599 (PMC13154922; doi:10.1002/smsc.202500599)
Supplement: Supplementary file 1 — Supplementary Material [file SMSC-6-e202500599-s001.zip › smsc70265-sup-0001-SuppData-S1.pdf]

Active particles in tunable compressible environments
Supplementary Information

Venkata Manikantha Sai Ganesh Tanuku,[1] Isha Malhotra,[2] Lorenzo Caprini,[2,3] Hartmut Löwen,[2] Thomas Palberg,[1] and Ivo Buttinoni[4]

[1] *Institute of Physics, Johannes Gutenberg University, 55128 Mainz, Germany*
[2] *Institute for Theoretical Physics II, Heinrich-Heine-Universität Düsseldorf, Universitätsstr. 1, 40225 Düsseldorf, Germany*
[3] *Present address: Sapienza University of Rome, Piazzale Aldo Moro 2, Rome, Italy*
[4] *Institute for Experimental Physics of Condensed Matter, Heinrich-Heine-Universität Düsseldorf, Universitätsstr. 1, 40225 Düsseldorf, Germany*

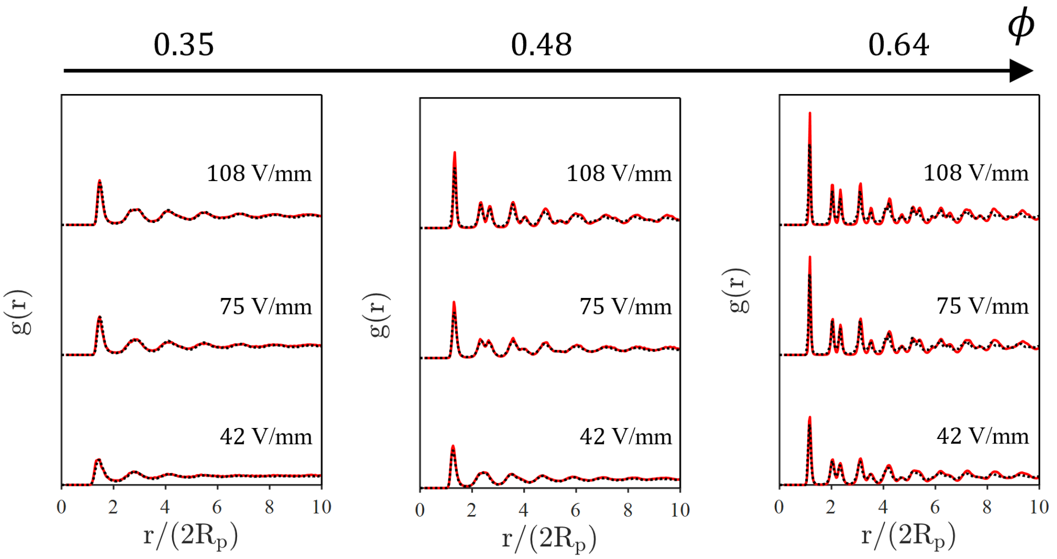

FIG. S1: Experimental (red curves) and numerical (dashed black lines) pair correlation functions, g(r), of passive colloidal monolayers of different packing fraction (ϕ, see arrow) subjected to various electric fields (E, indicated in the graphs). The radial distance is normalized by the particle diameter $2R_p$.

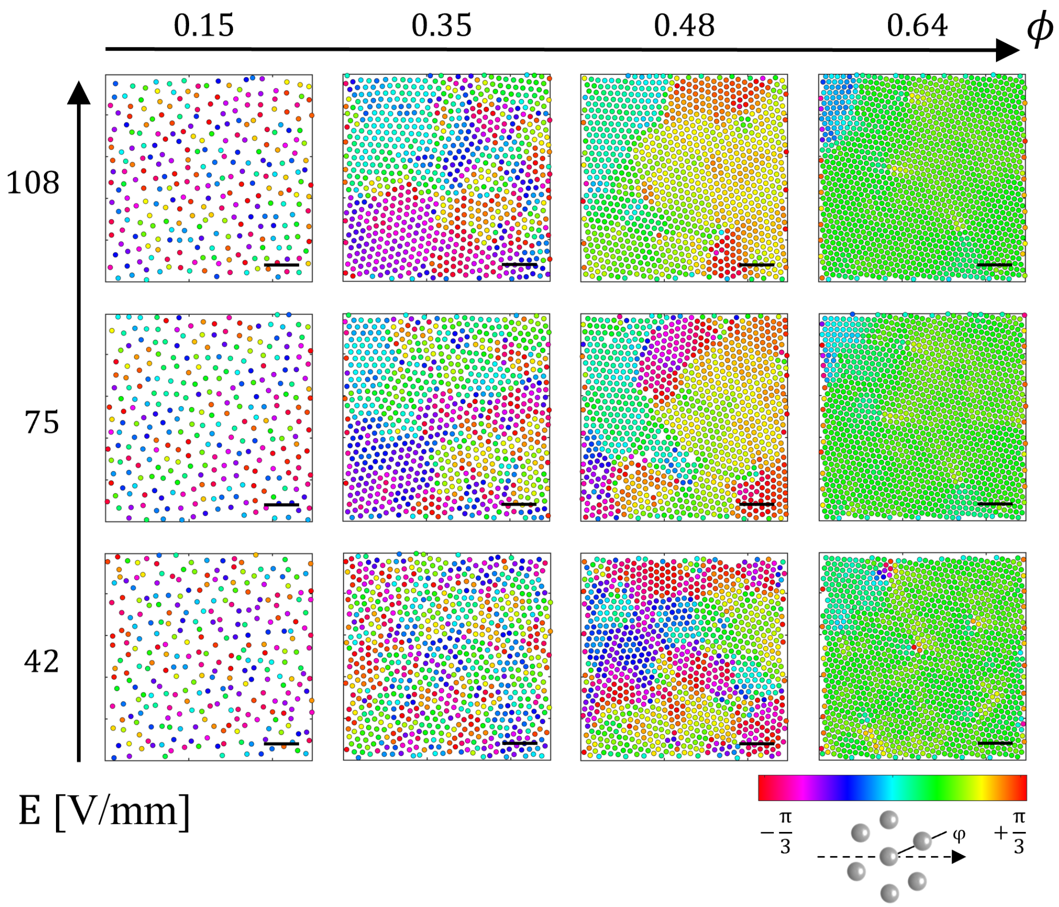

FIG. S2: Maps of the phase φ of the local hexagonal order parameter $\Psi_{6,\mathrm{n}}$ (where n is the particle label) corresponding to the last experimental frame of videos of passive colloidal monolayers of different packing fraction (ϕ, see horizontal arrow) subjected to various electric fields (E, see vertical arrow). The scale bar is 20 μm.

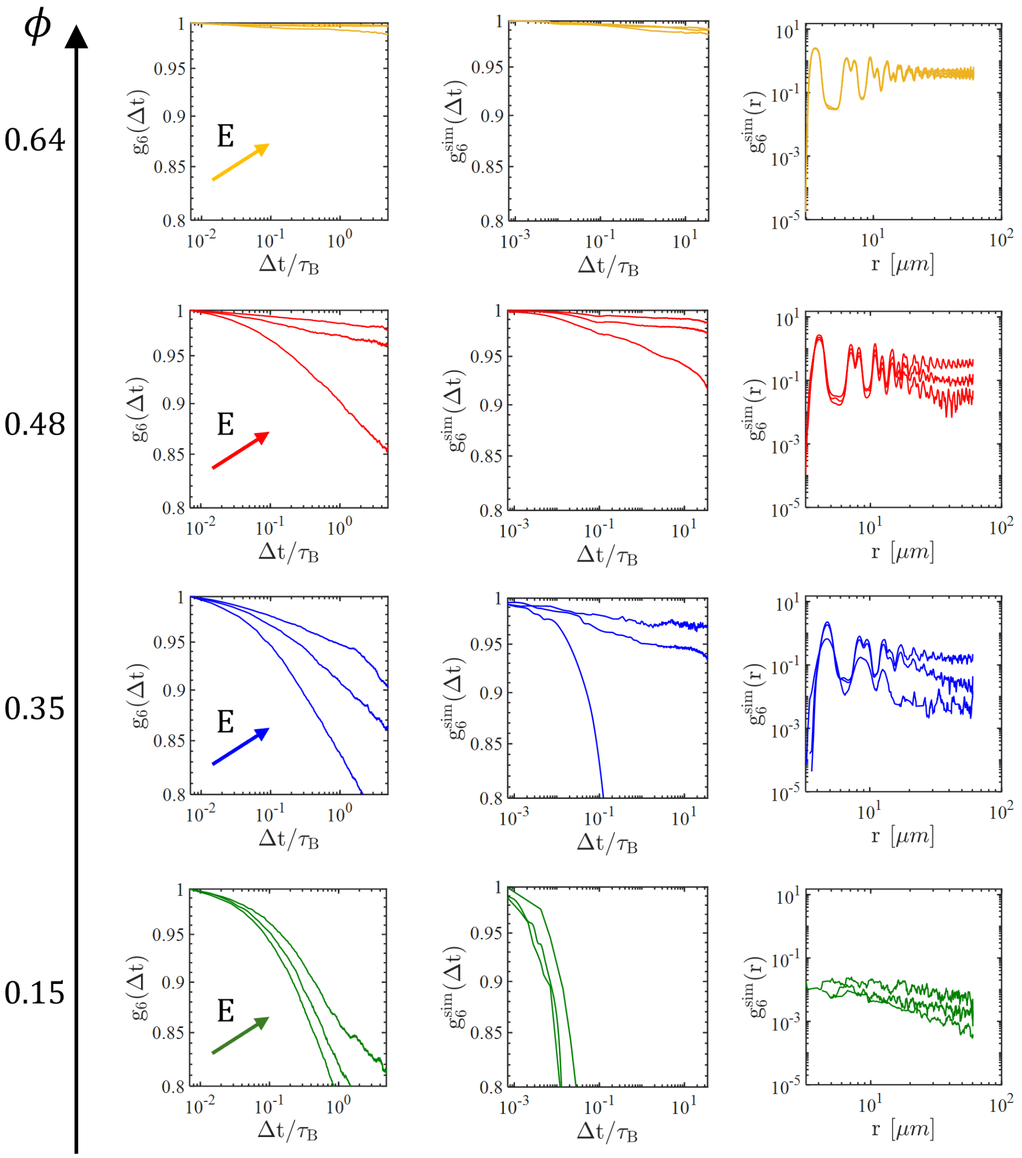

FIG. S3: Dynamical orientational correlation, g_6, plotted as a function of the normalized delay time $\Delta t/\tau_B$ (left and middle columns) and radial distance r (right column) for passive colloidal monolayers at different area fraction (ϕ, see vertical arrow). Right column: experimental $g_6(\Delta t)$. Middle column: numerical $g_6^{sim}(\Delta t)$. Right column: numerical $g_6^{sim}(r)$. As in Fig. 2 of the main text, we plot the curves at E = 48 V/mm, 75 V/mm and 108 V/mm using the same color; higher values of E lead to slower decays, as indicated by the coloured arrows. The experimental $g_6(r)$ is not accessible due to the limited field of view.

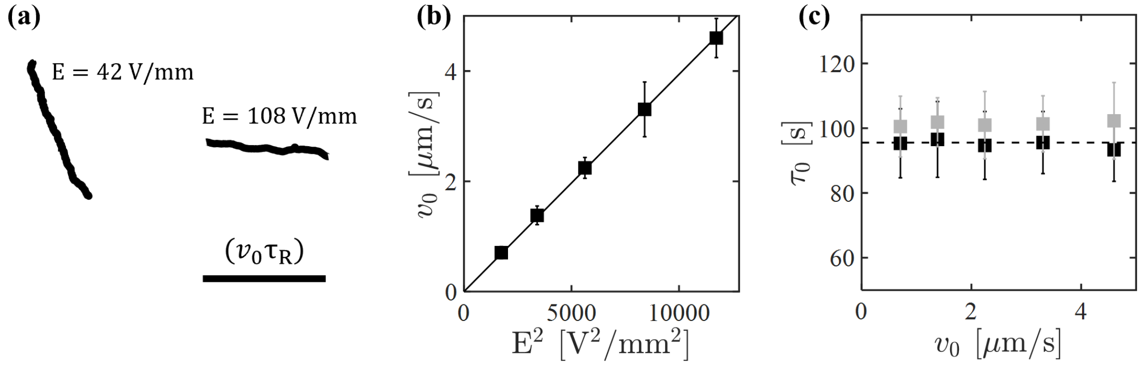

FIG. S4: (a) Trajectory of two free active particles actuated by AC fields of different strength. As in Fig. 3 of the main text, the scale bar correspond to the persistence length of particles cruising at mean velocity v_0 and undergoing rotational diffusion with timescale τ_R. (b) Mean swimming velocity as a function of the electric-field strength squared. (c) Characteristic persistence time τ_0 *vs.* E^2 estimated from the fits of the translational mean squared displacement (gray data) and the autocorrelation function of the velocity vector (black data, as in the main text). The horizontal dashed line marks the value of τ_R. In (b) and (c), the error bars are obtained by considering approximately 20 trajectories for each value of E.

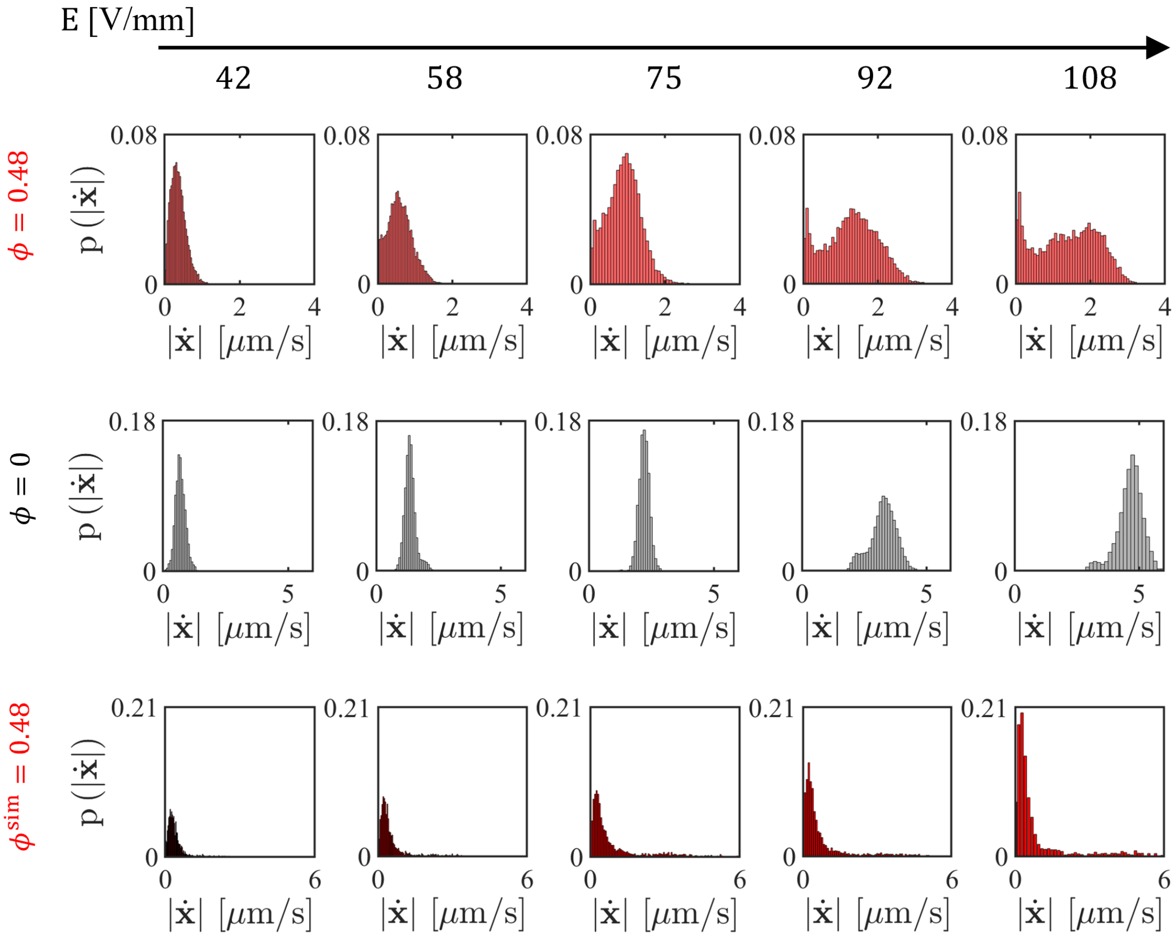

FIG. S5: Normalized histograms of the instantaneous velocities $|\dot{\mathbf{x}}|$ of approximately 20 active particles swimming throughout a colloidal monolayer at $\phi = 0.48$ (red histograms) and $\phi = 0$ (gray histograms) for five different values of E, as indicated by the arrow. The figure highlights the bimodal distributions observed in the colloidal bath, as opposed to the 'free' case ($\phi = 0$). Note that, for a given E, the mean swimming velocity of 'free' active particles is systematically larger.

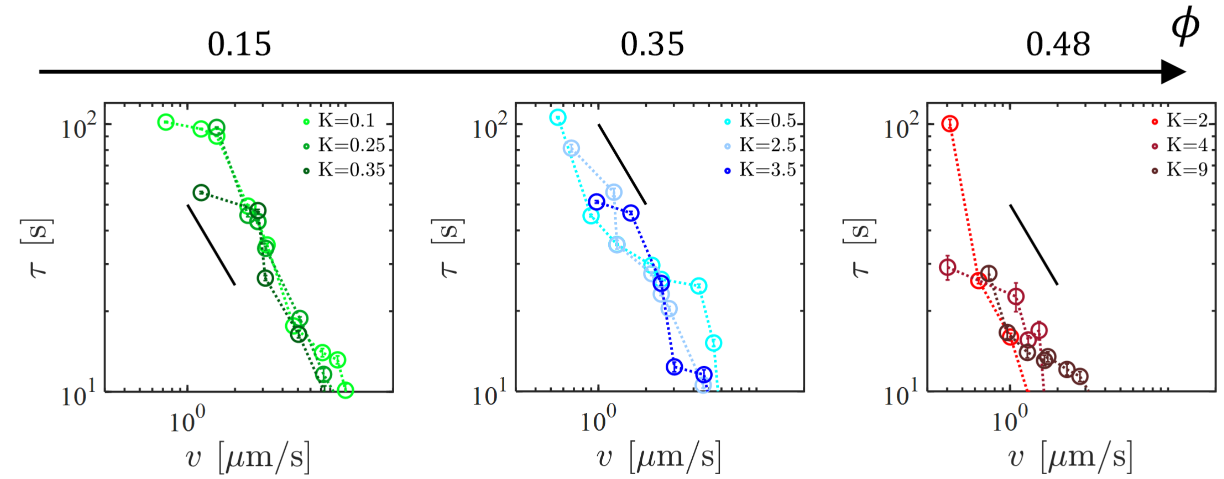

FIG. S6: Log-log plot of the numerical persistence time τ as function of the mean swimming velocity v in colloidal monolayers of different packing fraction (ϕ, see arrow). The colors correspond to the strength K of the dipolar interactions, which in these particular simulations does not depend on v. The solid black lines have slope -1, suggesting that the relationship $\tau \propto v^{-1}$ approximately holds for a broad range of packing fractions and interaction strengths.

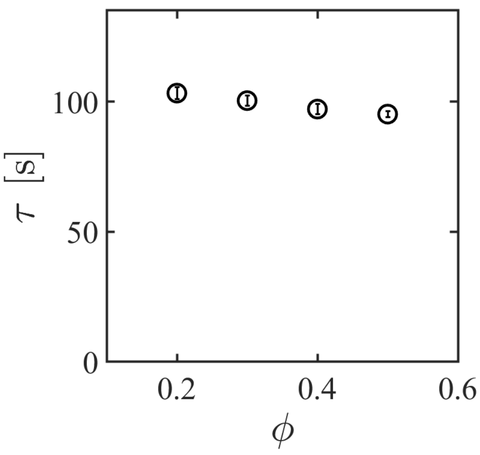

FIG. S7: Persistence time τ of Janus particles cruising at $v \approx 1.7$ μ/s in monolayers of passive particles interacting only via WCA potentials (K = 0). The data are from numerical simulations.

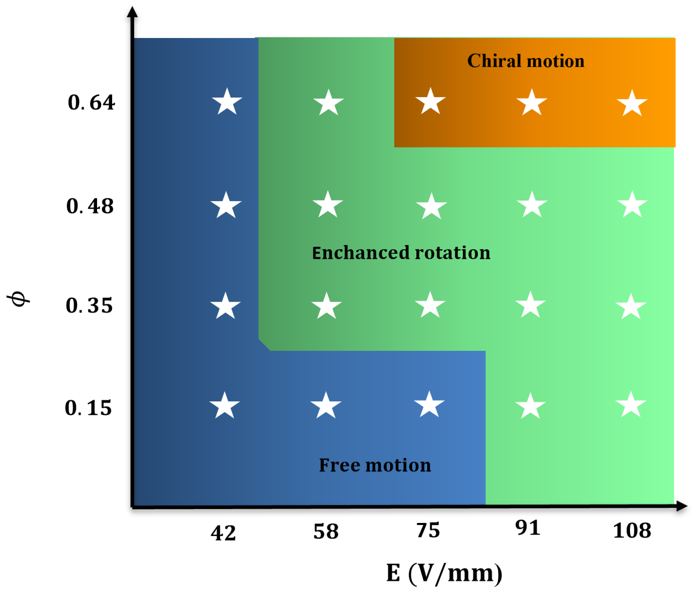

FIG. S8: Qualitative diagram showing the type of active motion observed as function of the applied electric field E and packing fraction ϕ of the environment. (blue) "Free motion" where $\tau \approx \tau_{\mathrm{R}}$. (green) "Enhanced rotation" where $\tau < \tau_{\mathrm{R}}$ but trajectories are not fully circular. (orange) "Chiral motion" where the active particles undergo helical motion with well-defined orbit's radius.

ϕ	E [V/mm]	K	ϕ	E [V/mm]	K	ϕ	E [V/mm]	K	ϕ	E [V/mm]	K
	42	0.10		42	0.5		42	2.0		42	11
	58	0.15		58	1.0		58	3.5		58	13
0.15	75	0.25	0.35	75	2.5	0.48	75	4.0	0.64	75	15.5
	92	0.28		92	3.0		92	5.0		92	15.5
	108	0.35		108	3.5		108	9.0		108	15.5

TABLE S1: Working parameters: for each packing fraction ϕ, we vary the applied electric-field magnitude E (in experiments) and the strength of the dipole-dipole interactions between SiO_2 particles (in simulations). K is determined by matching the numerical pair correlation functions with the experiments.

Supplementary Videos Description

- **Supplementary Video S1:** Active Janus particle cruising in a colloidal monolayer of Brownian microspheres ($\phi = 0.48$) under an applied electric field E = 42 V/mm. Recording speed: 2 fps. Playing speed: 100 fps. Scale bar: 30 μm.
- **Supplementary Video S2:** Active Janus particle cruising in a colloidal monolayer of Brownian microspheres ($\phi = 0.48$) under an applied electric field E = 108 V/mm. Recording speed: 2 fps. Playing speed: 100 fps. Scale bar: 30 μm.
- **Supplementary Video S3:** Structural evolution of a colloidal monolayer of passive silica spheres subjected to electric fields of different magnitude (as indicated in the legend). Recording speed: 10 fps. Playing speed: 200 fps. Scale bar: 20 μm.
- **Supplementary Video S4:** Free active Janus particle actuated by an applied electric field E = 42 V/mm. Recording speed: 10 fps. Playing speed: 100 fps. Scale bar: 30 μm.
- **Supplementary Video S5:** Free active Janus particle actuated by an applied electric field E = 108 V/mm. Recording speed: 10 fps. Playing speed: 100 fps. Scale bar: 30 μm.
- **Supplementary Video S6:** Intermittent motion of an active Janus particle swimming in a colloidal monolayer of Brownian microspheres ($\phi = 0.48$) under an applied electric field E = 92 V/mm. Recording speed: 2 fps. Playing speed: 30 fps. Scale bar: 30 μm.
- **Supplementary Video S7:** Active Janus particle cruising in a colloidal monolayer ($\phi = 0.48$) of Brownian microspheres of similar size (active particle radius $R_a = 2.5$ μm, passive particle radius $R_p = 2.1$ μm) under an applied electric field E = 108 V/mm.
- **Supplementary Video S8:** Active Janus particle undergoing helical motion in a colloidal monolayer of Brownian microspheres ($\phi = 0.64$) under an applied electric field E = 108 V/mm. Recording speed: 2 fps. Playing speed: 40 fps. Scale bar: 20 μm.
